# Human Mesenchymal Stromal Cell-Derived Extracellular Vesicles Modify Microglial Response and Improve Clinical Outcomes in Experimental Spinal Cord Injury

**DOI:** 10.1038/s41598-017-18867-w

**Published:** 2018-01-11

**Authors:** Katherine A. Ruppert, Tin T. Nguyen, Karthik S. Prabhakara, Naama E. Toledano Furman, Amit K. Srivastava, Matthew T. Harting, Charles S. Cox, Scott D. Olson

**Affiliations:** Department of Pediatric Surgery, McGovern Medical School at The University of Texas Health Science Center at Houston, Houston, TX USA

## Abstract

No current clinical intervention can alter the course of acute spinal cord injury (SCI), or appreciably improve neurological outcome. Mesenchymal stromal cells (MSCs) have been shown to modulate the injury sequelae of SCI largely via paracrine effects, although the mechanisms remain incompletely understood. One potential modality is through secretion of extracellular vesicles (EVs). In this study, we investigate whether systemic administration of EVs isolated from human MSCs (MSCEv) has the potential to be efficacious as an alternative to cell-based therapy for SCI. Additionally, we investigate whether EVs isolated from human MSCs stimulated with pro-inflammatory cytokines have enhanced anti-inflammatory effects when administered after SCI. Immunohistochemistry supported the quantitative analysis, demonstrating a diminished inflammatory response with apparent astrocyte and microglia disorganization in cord tissue up to 10 mm caudal to the injury site. Locomotor recovery scores showed significant improvement among animals treated with MSCEv. Significant increases in mechanical sensitivity threshold were observed in animals treated with EVs from either naïve MSC (MSCEv^wt^) or stimulated MSC (MSCEv^+^), with a statistically significant increase in threshold for MSCEv^+^-treated animals when compared to those that received MSCEv^wt^. In conclusion, these data show that treatment of acute SCI with extracellular vesicles derived from human MSCs attenuates neuroinflammation and improves functional recovery.

## Introduction

Acute spinal cord injury continues to be a devastating problem worldwide with high morbidity and mortality. Global incidence varied across sixty years from 8 to 246 cases per million each year^1–3^. Incidence in the US is 54 per 1 million annually, averaging approximately 17,500 new cases each year^4^. The incidence and prevalence of SCI is among the highest in the world, though the proportion of complete transections has been decreasing^5^. Mortality after hospital admission following acute spinal cord injury ranges from 4.4% to 16.7% globally^6^. Long term morbidity includes sensory, motor, and autonomic dysfunction.

While there are no effective pharmacological interventions, cell-based therapy has become an appealing alternative. Mesenchymal stromal cells (MSCs) have shown promising wide therapeutic potential in central nervous system trauma, including traumatic brain injury, stroke, and spinal cord injury. The effects of MSCs have been attributed to paracrine support of homeostatic conditions via immunomodulation, angiogenesis and cellular support, leading to recovery of function^7–17^. In SCI, engraftment of MSCs demonstrated significant therapeutic effects in pre-clinical studies^18–28^. The communication between stem and injured cells is important for the transmission of trophic signals, however, the details of these processes are not yet understood. One potential mechanism for intercellular exchange is through the secretion of vesicles.

MSCs also have a strong capacity for secretion of extracellular vesicles (EVs) in response to cellular injury^29^ and EVs isolated from MSCs exhibit stem cell-like regenerative activity^30^. EVs are small (40 nm-1 μm), heterogeneous particles with a lipid bilayer, containing growth factors, lipids, microRNAs, mRNAs, tRNAs, and proteins^31–33^. Although, their physiologic role is not well elucidated, EVs are suspected to participate in paracrine cellular communication^34^, provide trophic signals leading to tissue repair^29^, allow genetic exchange between stem and injured cells^35^, and attenuate inflammatory responses^35^. MSC-derived extracellular vesicles (MSCEv^wt^) can exhibit stem cell-like self-regenerative activity^36^, but potentially have decreased malignant potential, are less immunogenic, and evade the pulmonary first pass effect relative to MSC^37,38^.

Recently, we evaluated the immunomodulatory effects of EVs derived from inflammation-stimulated and naïve MSCs (MSCEv^+^ and MSCEv^wt^, respectively) using current Good Manufacturing Practice (cGMP)-compliant tangential flow filtration (TFF) system^39^. Differences in protein composition, cytokine profiles, and RNA content were discovered after detailed characterization of both MSCEv^wt^ and MSCEv^+^. MSCEv^+^ attenuated pro-inflammatory cytokine release *in vitro* compared to MSCEv^wt^, with different patterns of EV-uptake by activated primary leukocyte subpopulations. Overall, this investigation demonstrated that inflammatory-stimulated MSCs release EVs with enhanced anti-inflammatory properties, partially due to COX2/PGE2 pathway alteration.

*In vivo* studies have demonstrated potential therapeutic benefits of MSCEv therapy for both acute neurologic injury and neurodegenerative disorders^40^. Systemic delivery of MSCEv^wt^ in both mouse^41^ and rat models of traumatic brain injury (TBI)^42,43^ results in increased angiogenesis, neurogenesis^41^ and decreased neuroinflammation with concomitant functional recovery^42,43^. Therefore, we propose that MSCEv^wt^ and MSCEv^+^ intravenous treatment of spinal cord injured rats will result in significantly improved locomotor recovery, measured by Basso, Beattie, Bresnahan (BBB) Locomotor Rating, and improved mechanical sensitivity measured by the Dixon Up-Down Mechanical Threshold protocol when compared to vehicle-treated controls. We also hypothesize that treatment with MSCEv^+^ after SCI will result in additionally elevated locomotor and sensory recovery compared to those treated with MSCEv^wt^. Improvements in functional outcomes in the rat SCI model may be attributed to decreased neuroinflammation and attenuation of secondary injury mechanisms.

## Materials and Methods

### Isolation and culture of human mesenchymal stromal cells (hMSCs)

hMSCs were isolated from commercially available fresh human bone marrow aspirates of a 34 year old male (AllCells, Alameda, CA) using density centrifugation and plastic adherence as previously described^44^. An adherent population of MSCs was obtained 3 weeks after the initiation of culture. The cells were screened for typical spindle-like morphology and growth kinetics. The cells were further expanded by plating 10^6^ passage 2 cells at 200 cells/cm2 in 2528 cm² in Nunc™ Cell Factory™ Systems with complete culture medium (CCM) that consisted of α-minimal essential medium (α-MEM; Life Technologies, Grand Island, NY), 17% fetal bovine serum (FBS; Atlanta Biologicals, Norcross, GA), 100 units/ml penicillin (Life Technologies, Carlsbad, CA), 100 μg/ml streptomycin (Life Technologies, Carlsbad, CA), and 2 mM L-glutamine (Life Technologies). At 70% cell confluency, the medium was discarded, the cultures were washed with phosphate-buffered saline (PBS) (Life Technologies, Carlsbad, CA), and the adherent cells harvested with 0.25% trypsin (Life Technologies, Carlsbad, CA) for 5 min at 37 °C and frozen at 10^6^ cells/ml for subsequent experiments as passage 3 cells^14^. All hMSC used exhibited typical plastic adherence, morphology, and phenotype consistent with the ISCT consensus definition of “MSC” (negative for CD34, CD45, CD19, and HLA-DR, positive for CD44, CD73, and CD90)^45,46^.

To stimulate the naïve hMSCs, the cells were cultured in the CCM with TNF-α (20 ng/ml) + IFN-γ (20 ng/ml) inflammatory cytokines cocktail overnight. After which, the medium was removed, cells were washed with PBS and fresh serum-free CCM was added. After culturing for another 48 hours, the medium was collected and used for EVs isolation.

### Isolation of EVs by sequential filtration

EVs were isolated from both naïve (MSCEv^wt^) and stimulated MSCs (MSCEv^+^) as previously described^39^. We used large format cell culture flasks, and cultured hMSCs (1000 cells/cm^2^) to ~75% confluency over 5–6 days in 3180 cm2 Corning CellStack Systems (5 layer) to 500 ml serum-free CCM at a time by approximately 18–25 × 10^6^ hMSCs. After 48 hours of culture, the conditioned media was collected and pre-filtered through a 0.2 μm membrane to remove floating cells and cell debris, followed by a volume reduction and buffer exchange using tangential flow filtration (TFF) with a 300 k MW filter cassette (Millipore, Billerica, MA). This allowed large volumes of conditioned media into a concentrated, EV-enriched solution appropriate for use both *in vitro* and *in vivo*.

### Characterization of EVs

#### Particle size distribution and quantification by Nanoparticle tracking analysis (NTA)

EVs were characterized for particle size distribution and quantification by Nanoparticle Tracking Analysis (NTA), and expression of surface markers by flow cytometry, as previously described [paper under review]. Samples of extracellular vesicles were quantified via Brownian diffusion size analyses using ZetaView instrumentation (Particle Metrix, Germany). Sample aliquots were diluted 10^2^–10^6^-fold to achieve optimal concentration for analysis; 1.0 mL of diluted sample was used for each analysis. Light scattering of individual particles in solution was digitally recorded, particle trajectory and displacement was automatically analyzed by image analysis tracking software, and the particle-size distribution was determined from the observed Brownian motion of individual particles according to the Stokes-Einstein relationship. MSCEv’s are additionally screened for immunomodulatory activity using a cytokine inhibition assay^39^ for consistency in potency between preparations and donors, as well as the presentation of typical surface markers by flow cytometry.

### Flow cytometry analysis of rat tissue

Microglial isolation and analysis was performed as previously published^47^. In brief, microglia were isolated by first extracting the spinal cord segments at 14 days post-injury and then mechanical and enzymatic digestion was used to obtain a single-cell suspension using a Neural Cell Dissociation kit (Miltenyi Biotec). We utilized density centrifugation to remove a large amount of myelin, followed by a CD11b/c enrichment using a magnet activated sorting (MACS) kit (Miltenyi Biotec). The resulting myeloid-enriched cells were then stained for CD45CD11, P2Y12, CD32, CD86, CD200R, RT1B, CD163, and a Ghost™ viability dye (TONBO) with the addition of Cyto-Cal™counting beads (Thermo Fisher Scientific). This optimized multicolor immunofluorescence panel (OMIP) was designed to phenotype rat-derived microglia from spinal cord tissue after SCI, and allows for differentiation between macrophages and microglia, as well as phenotypic changes in microglia.

Blood samples were collected and spleens were harvested. Spleens were mechanically dissociated into single cells suspension. Briefly, spleens were placed in C tube (Miltenyi Biotec) and added with 10 ml of PBS. Using Spleen digestion program on the GentleMACS (Miltenyi Biotec), spleens were dissociated for 1 minute, washed twice with PBS and filtered with 70 µm cell strainer. From each sample of blood or spleen 100 µl were added with either lymphoid or myeloid antibody mix. The lymphoid multi-color panel is composed of CD3-FITC (Biolegend cat# 201403), CD25 -PE (Biolegend cat #202105), CD8a–PerCP (Biolegend cat # 201712), CD11b/c-PE Cy7 (BD Biosciences cat # 562222), RT1B-Alexa Fluor 674 (BD Biosciences cat # 562223), CD4-APC Cy7 (Biolegend cat# 201518), CD45RA-V450 (BD Bioscences cat # 561626). The myeloid multi-color panel is composed of His48–FITC (Invitrogen cat# 11057082), CD43-PE (Biolegend cat # 202812), CD8a–PerCP (Biolegend cat # 201712), CD11b/c-PE Cy7 (BD Biosciences cat # 562222), CD161-APC (Biolegend cat# 205606), CD4-APC Cy7 (Biolegend cat# 201518), CD172a-V450 (Biolegend, custom made). Cells were incubated for 20 minutes at room temperature and fixed in TQPrep™ Workstation (Beckman Coulter). Samples were run on Gallios (Beckman Coulter) to collect 10,000 events, and analyzed using Kalusa vr. 1.5a analysis software (Beckman Coulter).

### Animals

All protocols involving the use of animals were following the National Institutes of Health Guide for the Care and Use of Laboratory Animals and were approved by the University of Texas Health Science Center at Houston Animal Welfare Committee, with is the Institutional Animal Care and Use Committee. Rats were purchased from Envigo (Indianapolis, IN, USA) for use in this study. The animals were housed on a 12 h light/dark cycle with *ad libitum* access to food and water.

### Spinal Cord Contusion and EV treatment

Adult, male Sprague-Dawley rats (Envigo, Harlan, USA) weighing 225–250 g were utilized in this study. Animals were anesthetized with 4% inhaled isoflurane in oxygen and maintained using 1.5 L/min of 2–3% inhaled isoflurane in oxygen throughout the procedure. Animals received a laminectomy at spinal cord thoracic level 10 (T10), and the vertebral column was stabilized at T9 and T11. A moderate contusion injury (150 kdynes of force with 1 s dwell) was delivered to the spinal cord at T10 using the Infinite Horizon Impactor (Precision Systems and Instrumentation, LLC, Fairfax Station, VA), without opening the dura mater^48^. The overlying muscles were immediately sutured and the skin was closed with stainless steel wound clips. Beginning the day after SCI surgery, each animal’s urinary bladder was manually expressed twice daily until the animal recovered the ability to void its bladder. As a rule, bladder care was ceased when an individual animal exhibited an empty bladder on two consecutive bladder care sessions. To minimize post-surgical pain, animals were treated twice daily for five days post-SCI with buprenorphine (0.02 mg/kg s.q.). To prevent dehydration, animals also received a 3-ml bolus of 0.9% saline s.q. twice daily for the first 72 h after surgery. To prevent infection, animals also received antibiotics (0.01 mg/kg gentamicin i.p.) once daily for 10 days. Animals were housed in groups of two and maintained under normal veterinary care.

Animals were randomly selected to receive either vehicle (phosphate buffered saline solution) or MSCEv injection intravenously via tail vein 3 hrs after sham or spinal cord injury. MSCEv^wt^ and MSCEv^+^ were injected at a concentration of 1 × 10 ^9^ Ev/ml and a volume of 1 ml.

### Behavioral Testing

Longitudinal behavioral was assessed in two cohorts of animals (Sham n = 6, SCI + vehicle n = 6, SCI + MSCEv^wt^ n = 8, SCI + MSCEv^+^ n = 8). Description of behavioral tasks are described in detail below.

### Hind limb Motor Function

Hind limb locomotor function was assessed using the Basso, Beattie and Bresnahan (BBB) open field locomotor scale^49^. Before SCI surgery, animals were allowed to acclimate to the open field testing environment (a 40′′-diameter plastic wading pool) in groups of four for 10 minutes daily until they ceased to exhibit fear-associated behavior (e.g., crouching, cowering away from the examiner, vocalizing) and displayed signs of comfort (e.g., grooming, accepting treats from the examiner’s hand, exhibiting continuous locomotion and exploration). No further acclimation to the testing environment was performed.

BBB scores were assessed on days 1, 2, 3, 5, 7, 10 and 14 post-injury. Animals were placed into the open field and allowed to freely move about for a period of 4 minutes. Two blinded, independent observers scored each hind limb using a scoring sheet. Hind limb BBB scores were rated using an abbreviated 16-point BBB Open Field Rating Scale, and inter-rater reliability was confirmed. The abbreviated locomotor scoring scale was modified from the 21-point BBB Open Field Rating Scale by Basso, Beattie and Bresnahan^49^. Due to the brief period of study, the scale was abbreviated to focus on the subtle differences observed in the acute recovery period. In our previous studies of similar length, the observed recovery scores were consistently below 16, making this a reasonable level for abbreviation. In this study, the locomotor scores for both hind paws were averaged to produce one score per test session. Data were analyzed for normality using a Shapiro-Wilk normality test with a confidence interval of 95%. Values were also analyzed for skewness, with the calculated values being between −0.5 and 0.5, the distribution is considered approximately symmetrical. BBB scores were analyzed using a two-way repeated measures ANOVA (factors: experimental groups and time points with repeated measures on the second factor), followed by a Bonferroni post-test.

### Mechanical Sensitivity

Mechanical hypersensitivity was defined as a decreased withdrawal threshold when the hind paw was presented with a series of calibrated von Frey monofilaments (Stoelting, Wood Dale, IL), consistent with previous studies^50^. Animals were habituated to the testing environment for 10 minutes on day 14 post-injury. A series of filaments delivering calibrated amounts of force (2 g to 15 g) were applied using the Dixon “up-down” method, and 50% mechanical withdrawal threshold was determined as previously described^50,51^. Scores were analyzed using a paired Student’s t test; effectiveness of pairing was determined by calculating the Pearson correlation coefficient (r).

### Immunohistochemistry

Rats were anesthetized and spinal cord tissue was harvested following perfusion with PBS and 4% PFA 14 days post-injury (Sham n = 2, SCI + vehicle n = 4, SCI + MSCEv^wt^ n = 4, SCI + MSCEv^+^ n = 4). Coronal sections at 12 µm or sagittal sections at 40 µm were sectioned on a cryostat (Leica CM1860), adhered to glass slides and stained using standard staining protocol. Briefly, the sections were first rinsed with tris buffered saline (TBS) 1X (Thermo Fisher Scientific, PA) three times for 5 minutes each followed by a blocking and permeabilizing step for 1 hour with TBS 1X with 0.3% Triton X-100 and 10% normal goat serum (Jackson Immuno Research, PA) at room temperature. Then they were incubated in primary antibody solution of TBS 1X with 0.025% Triton X-100 and primary antibodies, anti-Iba-1, anti-GFAP, anti-doublecortin or anti-NeuN at 4 °C overnight. Slides were rinsed in TBS 1X three times for 10 minutes each then incubated in secondary antibody solution of TBS 1X with 0.025% Triton X-100 and secondary antibodies at 1:500 for 2 hours at room temperature. The sections were washed thrice with PBST for 10 minutes each after incubation with secondary antibodies. Slides were let dry at room temperature before they were cover-slipped with DAPI Fluoromoun-G (Southern Biotech, Birmingham, AL). Primary antibodies: microglia (Iba-1, 1:500, Wako-chem, Richmond, VA), astrocytes (GFAP, 1:500), regenerating neurons (Doublecortin, 1:300, Temecula, CA), mature neurons (NeuN, 1:500, Millipore Sigma, Billerica, MA). Secondary antibodies: 1:500 Alexa Fluor-488: and 1:500 Alexa Fluor-568:A11011, Invitrogen. Microscopic images were acquired with a Leica DM4000B fluorescence microscope.

### Exclusion Criteria

Injured subjects were excluded from the study if the actual force of impact to the spinal cord fell outside a range between 150 and 175 Kdynes^52^. In addition, any injured subjects receiving an impact of force within that range that exhibited a hind limb locomotor score greater than 2 on post-injury day 1 were also excluded^49^.

### Statistical Analysis

All data was analyzed using GraphPad Prism (GraphPad Software, Inc., La Jolla, CA) using an analysis of variance (ANOVA) with Tukey post-hoc analyses for multiple comparisons. Values of p < 0.05 were considered significant. All group data is presented as mean +/− standard error or standard deviation. Sample sizes were determined *a priori* using G*Power Statistical Power Analyses Software^53^.

### Data Availability

The datasets generated during and/or analyzed during the current study are not publicly available due to inclusion in pending publications but are available from the corresponding author on reasonable request.

## Results

### MSCEv Attenuate Microglia Activation Pathways

**Figure 1:**
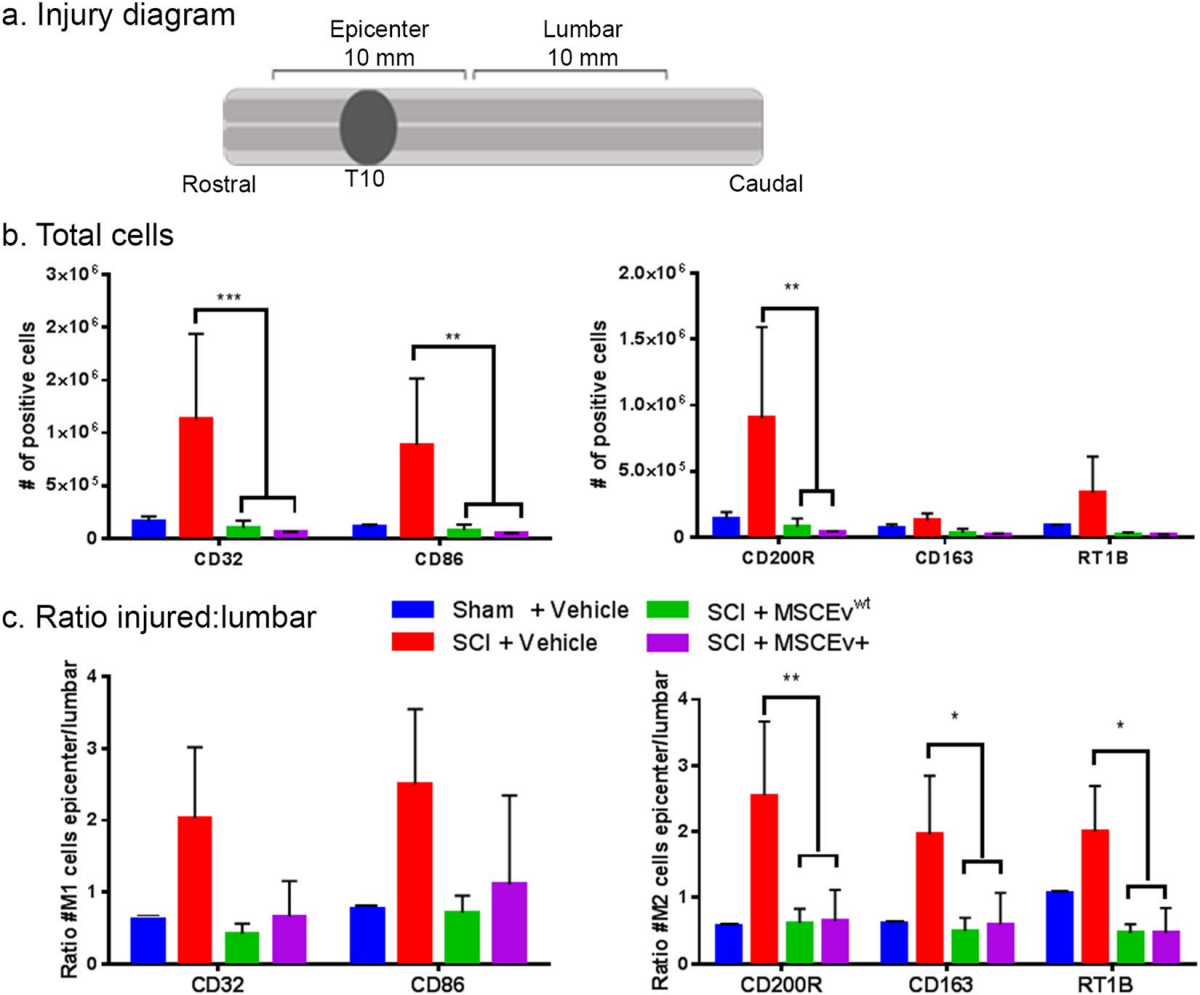
Microglia activation in injured spinal cord Samples of spinal cord tissue containing the injury epicenter (**a**) were analyzed by flow cytometry for microglia activation state phenotypes. Interestingly, the total number of cells positive for markers associated with pro-inflammatory, M1 microglia (CD32 and CD86) were significantly decreased after treatment with MSCEv^wt^ and MSCEv^+^ (***p < 0.001, **p < 0.01, respectively)(**b**). Additionally, lumbar sections of spinal cord were analyzed, revealing the ratio of positive cells in injured sections of spinal cord and distal lumbar sections of spinal cord. Cells positive for M1 associated markers CD32 and CD86 are increased after SCI but treatment with either MSCEv^wt^ or MSCEv^+^ decrease levels of these cells to near sham values. Cells positive for M2 associated markers CD200R, CD163 and RT1B are significantly decreased (**p < 0.01, *p < 0.05, *p < 0.05, respectively) with treatment of either MSCEv^wt^ or MSCEv^+^ after SCI. Overall, there is a significant reduction in the number of activated microglia after treatment with either MSCEv^wt^ or MSCEv^+^, indicating that both EVs are effective in reducing inflammation in the injury epicenter as early as 14 days post-injury (**c**). Sham + Vehicle, n = 2, SCI + Vehicle, n = 2, SCI + MSCEv^wt^, n = 3, SCI + MSCEv^+^, n = 3.

Samples of spinal cord tissue containing the injury epicenter (Fig. 1a) were analyzed by flow cytometry for microglia activation phenotypes. Interestingly, the total number of cells positive for markers associated with pro-inflammatory, M1 microglia (CD32 and CD86) were significantly decreased after treatment with MSCEv^wt^ and MSCEv^+^ (***p < 0.001, **p < 0.01, respectively) when compared to those that were injured and received vehicle injections (Fig. 1b). Similarly, cells positive for M2 associated markers CD200R, CD163 and RT1B are significantly decreased (**p < 0.01, *p < 0.05, *p < 0.05, respectively) with treatment of either MSCEv^wt^ or MSCEv^+^ after SCI (Fig. 1b).

Lumbar sections of spinal cord were processed and analyzed. The ratio between the epicenter and the distal lumbar was calculated and was shown to be coherent with the marker expression by showing similar trend of significant reduction following treatment. (Fig. 1c). These results imply that the inflammation caused by the injury is local and that the MSCEv given are able to influence/target the inflammatory site and act locally as well. The expression of M1 associated markers in the CNS, p2y12, is a microglia specific marker. By assuming that microglia are CD11^+^CD45^+^ and p2y12^+^, we were able to extract data and apply this not only to microglia but also to the myeloid cells as the monocytes/macrophages, which we assumed to be CD11^+^CD45^+^p2y12^−^. This analysis revealed that both MSCEV and MSCEV + treatment reduces the expression of RT1B in the monocytes/macrophages (CD11b/c+, CD45+, P2y12−) populations in the spinal cord (Supplemental Figure S1). This data indicates that EVs are effective in reducing neuroinflammation in the injury epicenter as early as 14 days post-injury. SCI + Vehicle, n = 3, SCI + MSCEv^wt^, n = 3, SCI + MSCEv^+^, n = 3.

### MSCEv treatment following SCI results in improved locomotor recovery

**Figure 2:**
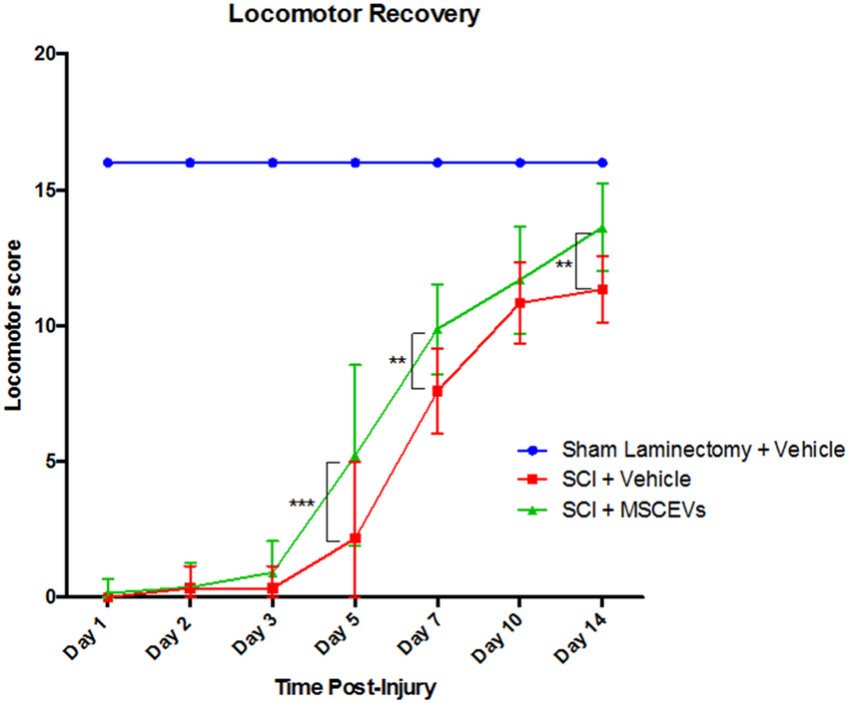
Animals treated with MSCEVs displayed significantly higher locomotor recovery scores when compared to sham and untreated SCI animals. At 5, 7 and 14 days post-injury, SCI + MSCEvs animals had significant improvement compared to untreated SCI (***p < 0.001, **p < 0.01, **p < 0.01, respectively). Treatment with MSCEVs results in significant functional improvement as early as 5 days following SCI. A linear regression was performed for both SCI + Vehicle and SCI + MSCEVs data (not shown). An increase in positive slope associated with SCI + MSCEVs compared to that of SCI + Vehicle indicates an increased rate of recovery (m = 1.1773, m = 1.0385, respectively). All data represented are means +/− SD. Sham + Vehicle, n = 6, SCI + Vehicle, n = 6, SCI + MSCEvs, n = 16. Data was statistically analyzed using Two-Way ANOVA with Tukey’s multiple comparison test.

Animals received either sham or T10 contusion SCI and were treated with MSCEv (MSCEv^wt^ and MSCEv^+^ groups combined) 3hrs after injury. All animals were observed for locomotor recovery using the BBB scale on days 1, 2, 3, 5, 7, 10 and 14 post-injury. SCI + MSCEv animals showed significant improvement in locomotor recovery scores on days 5, 7, and 14 post-injury vs. SCI rats treated with vehicle injections. SCI rats treated with MSCEv scored significantly higher on locomotor recovery assessments compared to untreated animals on post-injury day 5 (***p < 0.001), 7 (**p < 0.01) and 14 (**p < 0.01). (Fig. 2). Sham, n = 6, SCI + Vehicle, n = 6, SCI + MSCEv, n = 16. All data represented are means +/− SD. Data was statistically analyzed using Two-Way ANOVA with Tukey’s multiple comparison test. Raw data is included in Supplemental Table 1.

### Mechanical Sensitivity Threshold Improved with MSCEv

**Figure 3:**
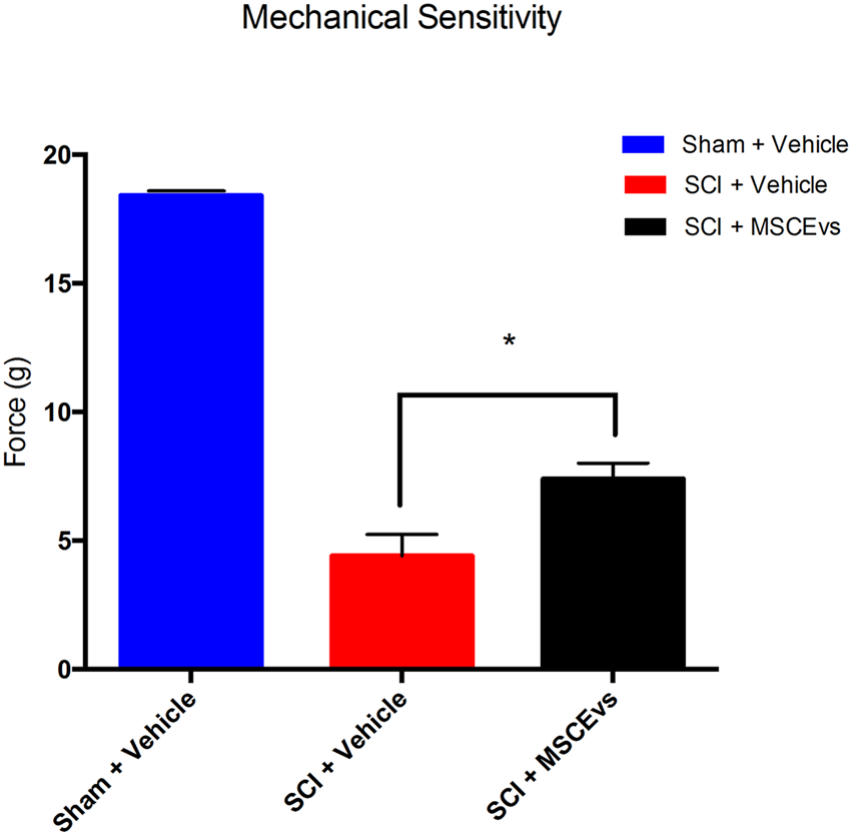
Animals treated with MSCEvs exhibited significantly higher force threshold (*p < 0.05) than those treated with vehicle when tested with the Dixon Up-Down method for mechanical sensitivity in the hind paws. All data was analyzed for statistical significance using One-Way ANOVA with Sidak’s test for multiple comparisons. Sham + Vehicle, n = 6, SCI + Vehicle, n = 6, SCI + MSCEvs, n = 16. Data represent mean values +/− SEM.

In addition to improved locomotor recovery, we observed a significant change in mechanical hypersensitivity after injury. Two weeks after injury we used calibrated von Frey filaments and the Dixon Up-Down scoring method to identify allodynia in hind paws and determine mechanical sensitivity thresholds. SCI animals treated with MSCEv (MSCEv^wt^ or MSCEv^+^) exhibited significantly higher force threshold (*p < 0.05) than those treated with vehicle when tested with the Dixon Up-Down method for mechanical sensitivity in the hind paws. Sham, n = 6, SCI + Vehicle, n = 6, SCI + MSCEv, n = 16. All data represented are means +/− SD. Data was statistically analyzed using One-Way ANOVA with Sidak’s test for multiple comparisons. (Fig. 3).

### Treatment with MSCEv^+^ from Inflammation-Stimulated MSC Improves Sensory Function

A cohort of SCI animals received injections of extracellular vesicles which were derived from MSC that we stimulated with inflammatory agents. EVs secreted by inflammation-stimulated MSC were then referred to as activated extracellular vesicles (MSCEv^+^).

**Figure 4:**
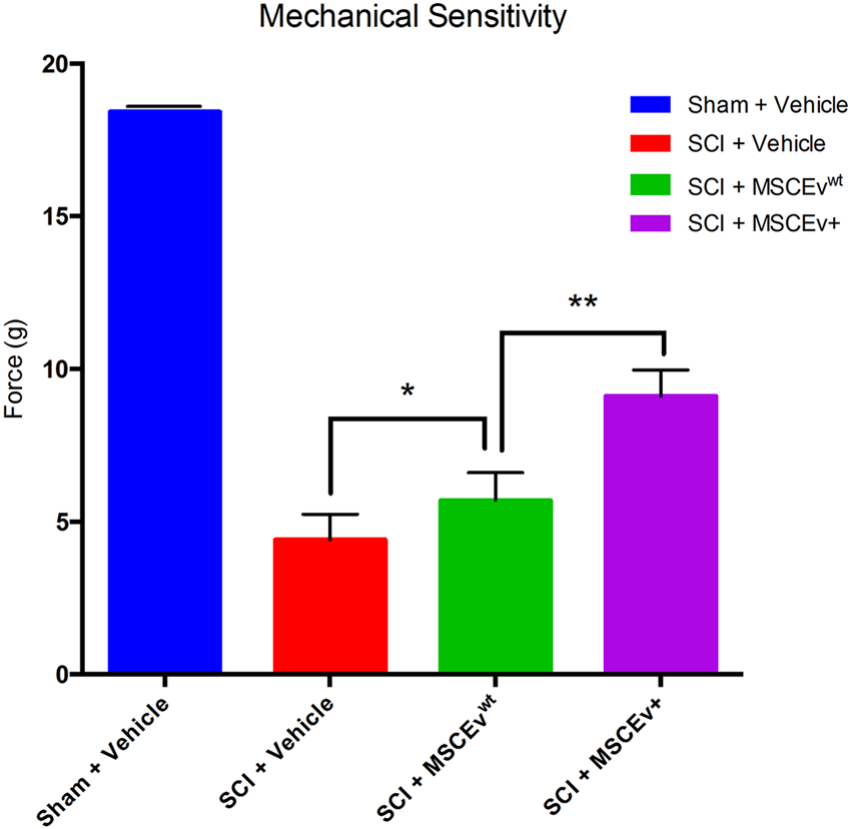
Animals treated with MSCEv^+^ exhibited significantly higher force threshold (**p < 0.01) than those treated with MSCEv^wt^ when tested with the Dixon Up-Down method for mechanical sensitivity in the hind paws. There was also a significant increase in threshold when treated with MSCEv^wt^ (*p < 0.05), when compared to untreated SCI animals. All data was analyzed for statistical significance using One-Way ANOVA with Sidak’s test for multiple comparisons. Sham + Vehicle, n = 6, SCI + Vehicle, n = 6, SCI + MSCEv^wt^, n = 8, SCI + MSCEv^+^, n = 8. Data represent mean values +/− SEM.

Animals treated with MSCEv^+^ exhibited significantly higher mechanical force threshold (**p < 0.01) than those treated with MSCEv^wt^ when tested with the Dixon Up-Down method for mechanical sensitivity in the hind paws. There was also a significant increase in threshold when treated with MSCEv^wt^ (*p < 0.05), when compared to untreated SCI animals. MSCEv^+^ displayed an increased therapeutic effect which can be attributed to the enhanced anti-inflammatory properties of MSCEv^+^ derived from inflammation-stimulated MSCs. Sham + Vehicle, n = 6, SCI + Vehicle, n = 6, SCI + MSCEv^wt^, n = 8, SCI + MSCEv^+^, n = 8. Data represent mean values +/− SEM. All data was analyzed for statistical significance using One-Way ANOVA with Sidak’s test for multiple comparisons (Fig. 4).

### MSCEv Effects on Immune Response in Blood and Spleen

**Figure 5:**
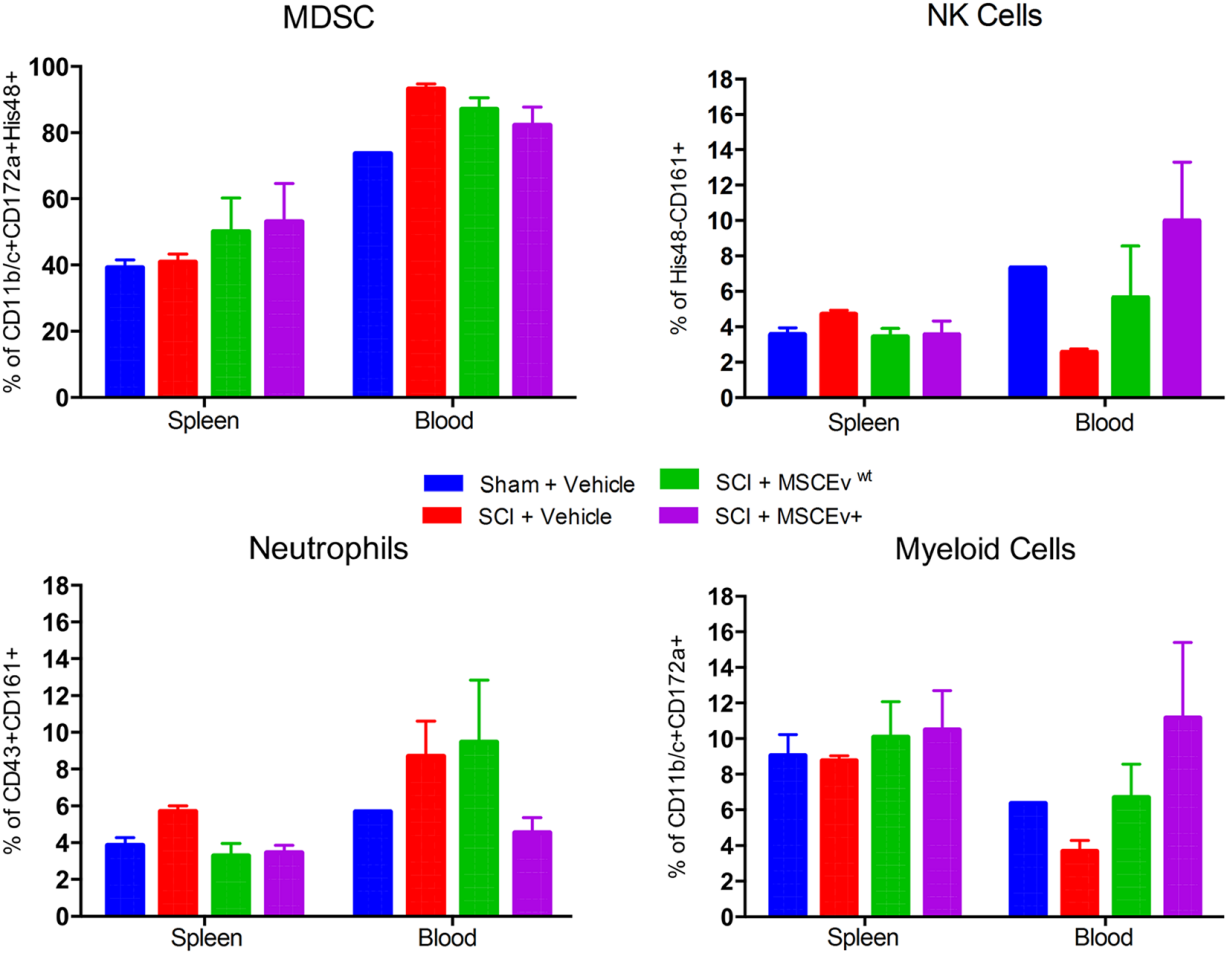
Flow cytometry of spleen tissue and blood samples from each animal provide evidence of anti-inflammatory effects of MSCEv^wt^/MSCEv^+^ treatment. Animals treated with either MSCEv^wt^ or MSCEv^+^ after SCI show approximately 5–10% decrease in MDSC present in the blood with an increase of approximately 10% in the spleen. This suggests that MSCEv^wt^/MSCEv^+^ treatment causes a retention of MDSCs in the spleen that normally migrate to the blood after SCI. The opposite appears to occur with NK cells. MSCEv^wt^ and MSCEv^+^ treatment both result in a decrease of NK cells in the spleen following SCI but restore blood levels of NK cells to sham values. Both treatments also resulted in a decrease in splenic neutrophils, however, MSCEv^+^ caused a decrease in blood neutrophils to sham-like values. Both treatments recover myeloid cells that are decreased in both spleen and blood following SCI. Sham + Vehicle, n = 2, SCI + Vehicle, n = 2, SCI + MSCEv^wt^, n = 3, SCI + MSCEv^+^, n = 3.

Using flow cytometry we were able to evaluate lymphoid and myeloid populations in the blood and spleen. The analysis of the myeloid population at 14 days after injury did not show significant changes in the spleen, and seem to be high in all the groups (Fig. 5). However, changes remain visible in the blood; treatment with MSCEv resulted in myeloid cell expansion that persists, while the injured group showed overall reduction in myeloid cells in circulation. Several subpopulations, such as neutrophils, NK cells and granulocyte, were analyzed. Neutrophils (CD43^+^CD161^+^) seemed to increase upon injury and remain high after 14 days, in both spleen and blood. Particularly in the blood, treatment with MSCEv^wt^ resulted in elevated levels of Neutrophils as well. NK cells (His48^−^CD161^+^) seem to remain at a range of 3–10% in both spleen and blood. Another subpopulation of granulocytes, often refer to as Myeloid-derived suppressor cells (MDSC), which were identified as CD11+CD172+His48+, seemed to expand upon MSCEv treatment in both spleen and blood.

In addition, we evaluated dendritic cells and T cells activation at 14 days. MSCEv groups showed increased levels of dendritic cells (DCs), compared to injured group (Supplemental Figure S2). T cell expansion was not significant between the groups at 14 days. The T cell/DC conjugate is a measurement for activation of the immune system. Indeed, the levels of the conjugates increased following MSCEv treatment, however these results are not significant, but do imply the safety of the treatment. No particular T cell subset expansion was observed. Sham + Vehicle, n = 3, SCI + Vehicle, n = 3, SCI + MSCEv^wt^, n = 3, SCI + MSCEv^+^, n = 3.

### MSCEv Decreases Reactive Microglia and Astrocytes

**Figure 6:**
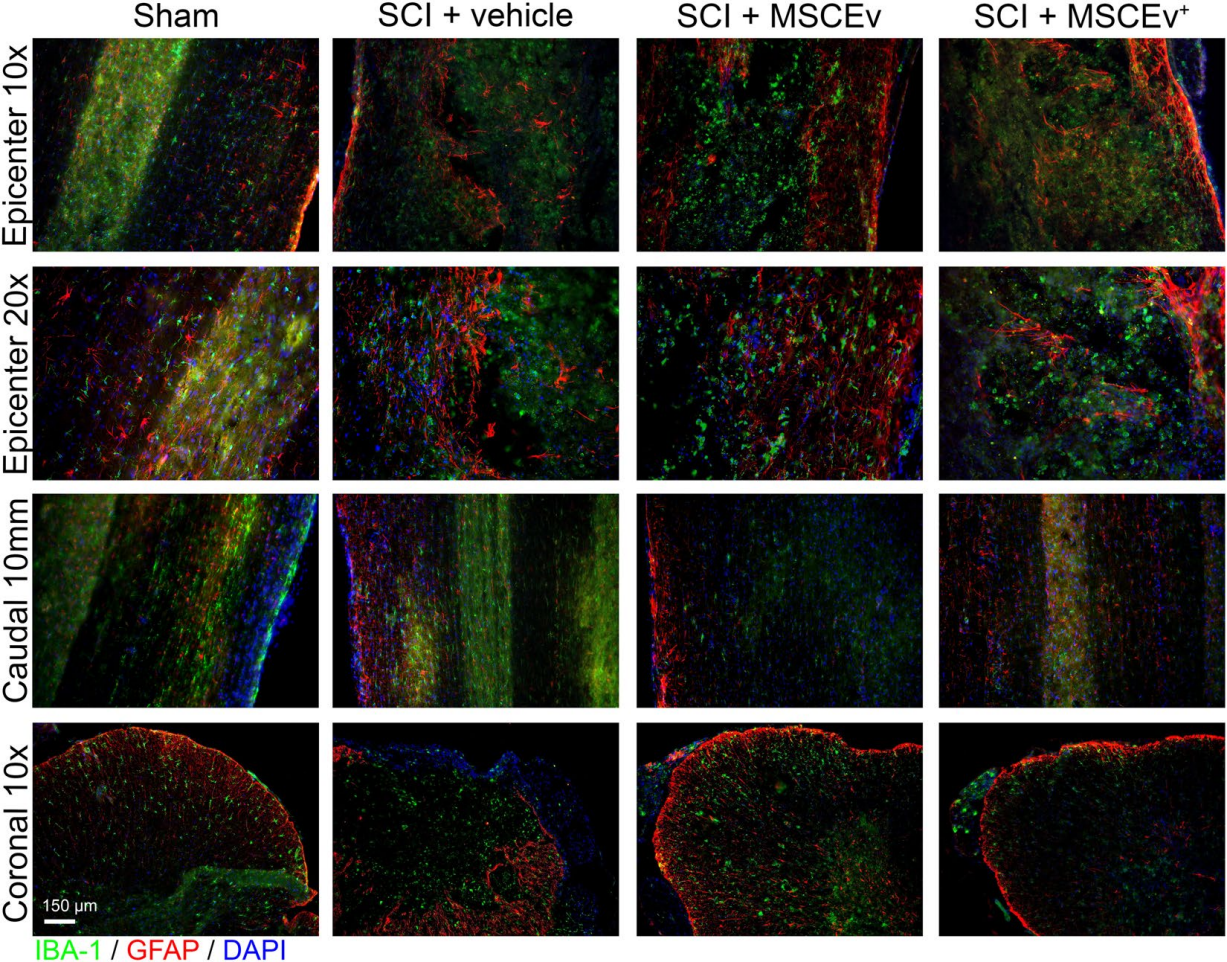
Longitudinal and coronal sections of spinal cord at 14 days post-injury were stained for Iba-1 (microglia, green), GFAP (astrocytes, red), and DAPI (nuclei, blue) and analyzed for neuroinflammation. Longitudinal sections were 40 μm in thickness while coronal sections were 12 μm thick. Representative images of the injury epicenter and a region 10 mm caudal to the epicenter are displayed above. Qualitative analysis revealed increased activation of microglia and astrocytes in injured tissues that appear to be less abundant in those from MSCEv^wt^ and MSCEv^+^ animals. Areas 10 mm caudal to the epicenter also exhibited activation of microglia and astrocytes, however, the decrease in activation is more noticeable in sections from MSCEv^wt^ and MSCEv^+^ treated animals. In coronal sections, prominent reactive astrocytes and microglia are visible in vehicle-treated animal tissues while there is much less reactivity in MSCEv^wt^ treated animal tissues, which is especially profound in the tissue of MSCEv^+^ treated animals. Sham + Vehicle, n = 2, SCI + Vehicle, n = 2, SCI + MSCEv^wt^, n = 3, SCI + MSCEv^+^, n = 3.

In examining representative immunohistochemical images of the epicenter and caudal cord, we noted a relative increase in GFAP signal intensity that directly corresponded with the injury. This increase in GFAP is consistent with the gross anatomical damage associated with spinal cord injury. In reference to coronal sections (Fig. 6), there is a relative loss of structure that is observed in comparing sham images to injured images. Corresponding images from treated animals appear more similar to the sham images. There also appears to be slight, relative variation in Iba-1-labeled structures in injured sections that do not resemble those of sham sections. It is not clear based on limited histology alone if treatment had an effect on these organizational changes, however the differences suspected in astrocyte and microglia may be associated with scar tissue formation. Sham + Vehicle, n = 2, SCI + Vehicle, n = 2, SCI + MSCEv^wt^, n = 3, SCI + MSCEv^+^, n = 3. (Fig. 6).

## Discussion

Our data demonstrate that MSCEv^wt^ and MSCEv^+^ attenuate inflammation and improve functional outcome in a rodent model of SCI. In this study, we utilized a cGMP-compliant system to isolate MSC-derived EVs from normal and inflammatory cytokine-stimulated cultures, which were then delivered intravenously 3 hr after T10 spinal contusion injury. Treatment with MSCEv resulted in an improvement in locomotor recovery, decrease in hyper-sensitivity, and an overall decrease in neuroinflammation. This effect persisted at least 14 d after treatment and is accompanied by differences in the myeloid and lymphoid profile of the blood and spleen. In most circumstances, the MSC-derived EV had no distinguishable differences, with the exception of hypersensitivity, where EV from inflammation-stimulated MSCs were able to significantly improve the treatment effect. Overall, MSCEv were successful in improving outcome after SCI.

Cellular therapies to treat SCI and other CNS injuries have been tested in many experimental animal models and clinical trials, however, the success of these studies is limited. While some studies explore stem-cell based cell and tissue replacement strategies, many studies target therapeutic intervention on secondary injuries in an attempt to limit cell death, spare white matter, and modulate immune responses and neuroinflammation to improve functional recovery. These studies, while promising, often have a number of technical hurdles to overcome and formidable challenges when applied at scale.

Our lab recently developed a multi-parameteric panel to analyze microglia using flow cytometry. This technique can differentiate between macrophages and resident microglia, quantify total cell numbers, as well as phenotypic changes to microglia polarization in rat models of TBI and SCI. Details of this technique can be found in Toledano-Furman *et al*.^47^. We observed a number of significant differences in putative pro-and anti-inflammatory populations, although the current understanding of rat CNS immunology has not fully matured. While much work has been devoted to characterizing mouse microglia markers, phenotypic characterization of rat microglia is not yet standardized, resulting in some difficulties in comprehensive data analysis. Our results show a significant decrease in the activation markers, which suggest overall attenuation of the inflammation at the injury site after MSCEv^wt^ or MSCEv^+^ treatment. This also implies that MSCEv may be capable of preventing a large neuroinflammatory response altogether rather than skewing microglial polarization. Tissue level neuroinflammation has been linked to both locomotor activity and mechanical sensitivity^54,55^.

Here, we show that animals treated with MSCEv have significantly improved locomotor recovery as early as 5 days post-injury (Fig. 2; *p < 0.001), with increasing significance at days 7, 10, and 14 post-injury when compared to vehicle-treated or MSC-treated SCI animals. In previous studies by our group and others, MSC-treated rats have shown increased threshold to mechanical stimulus and an improvement in locomotor function. In comparing our internal data sets, MSCEv recover locomotor function earlier than those treated with other cellular therapies in parallel studies (data not shown). In addition to locomotor recovery, MSCEv and particularly MSCEv^+^ were able to decrease mechanical sensitivity 14 d after injury.

In the presence of inflammatory stimuli, immune cells are differentially activated^56^. Activation causes a number of notable changes in marker expression, as well as proliferation. To better capture the global changes, we quantified cell numbers in addition to profiling marker expression. Evaluating different components of the cellular arm of the immune system revealed overall moderate changes, of which the majority were not significant with limited replicates. At the endpoint of the study, MSCEv treatment resulted in non-significant myeloid cell expansion. Injury resulted in elevated levels of neutrophils in both the spleen and blood. Treatment with MSCEv^+^, but not MSCEv^wt^ reduced the levels of these cells. Expansion of NK cells and granulocytes was observed in the blood. This granulocytic population was found to have immunosuppressive effects on T cells and macrophages. Saiwai *et al*.^57^, demonstrated the function of these myeloid cells in a mouse model of SCI and stated that they significantly contribute to attenuate acute inflammation after SCI.A similar trend was published by Wang *et al*. in a mouse model of SCI, where 3 days post-injury, blood myeloid cells were increased and maintained high level for at least 28 days post-injury^58^. Spleen myeloid were also increased on day 3, but rapidly decreased with time. In our study, MSCEv treatment resulted in a reduction in blood myeloid while spleen MDSC increase compared to controls. This result may indicate that MSCEv prevent egress of myeloid cells from the spleen as a method of reducing inflammation. Finally, the different subsets of T cells were evaluated, of which T cytotoxic were significantly increased in the circulation following treatment with MSCEv. The percent of T cell/DC conjugate was increased with treatment, but again, not significantly at that time point. These results were as expected, as systemic treatment with EVs was previously found to result in T cell function modulation^59^. T regulatory cells increased in the spleen but not in the blood fraction tested. Those results are coherent with previously published work demonstrating elevated levels of T regulatory following MSC exosomes systemic injection^60^. Overall we concluded that MSCEv treatment modulated the host immune response, at 14 days after injury the cellular components of the immune system support inflammation attenuation and thus tissue regeneration.

Extracellular vesicles (EV) secreted from MSC are of great interest as a new, cell-free therapeutic. To date, there are few studies of MSCEv used to treat CNS trauma, most of which focus upon TBI or stroke using normal or genetically modified MSC. In this study, we investigate whether systemic administration of EVs isolated from MSCs has the potential to be exploited as an alternative to cell-based therapy for SCI. EVs from MSCs were isolated using a current Good Manufacturing Practice (cGMP)-compliant tangential flow filtration system. Isolated EVs were characterized for the expression of surface markers (CD9, CD63, CD81 and CD107a) by flow cytometry and screened for the ability to reduce cytokines and reduce donor-to-donor and batch-to-batch variability. The ZetaView Nanoparticle Tracking Analyzer was used to determine the size distribution and quantitation of the EVs.

MSCEv^wt^ administered 24hrs after TBI in rodent models are reported to significantly improve spatial learning in Morris water maze approximately one month after injury and treatment^41,43,61^, as well as significantly reduce neurological deficits and foot-fault frequency 2–4 weeks after injury and treatment^43^. In experimental models of stroke, EVs from MSCs with overexpressed microRNA 133b have improved functional recovery and neuroplasticity compared to rats treated with PBS or control MSCEv^wt^, possibly due to secondary release of EVs from astrocytes^62^ downregulating connective tissue growth factor (CTGF)^63^. In these studies, and in work from other fields, MSCEv have been observed to be capable of replicating some or all of the therapeutic actions attributed to MSC. This includes the capacity to modulate the immune response without acting as a broad immunosuppressant or a traditional anti-inflammatory drug, like methylprednisone. The presence of numerous potential mechanisms of action, even in isolated MSCEv, suggests that it will be difficult to recapitulate their actions through single or combinatorial pharmaceutical treatments.

A single, recent study describes the therapeutic effects of EV from rat MSC on SCI-induced apoptosis, inflammation and angiogenesis. The authors delivered rat MSC-derived EVs systemically 30 minutes after T10 weight-drop contusion and reported a decrease in apoptosis and inflammatory cytokines, improved locomotor recovery, and promoted angiogenesis^64^. Locomotor recovery in our study supersedes that of BBB scores of SCI + EV animals reported in the literature^64^, with higher BBB scores at each time point and statistical significance achieved starting 5 days after injury, which is 9 days prior to significance achieved in the Huang *et al*., study. This difference, while notable, could be explained by either differences in EV potency or differences in experimental methodology. Importantly, our study utilizes cGMP-compatible techniques and reagents to create scalable preparations of human MSC-derived extracellular vesicles in an effort to facilitate and streamline translation to clinical applications.

In this study, we delivered MSCEv 3 hrs post-injury, primarily to translate to real world applications. Specifically, it would be logistically challenging to deliver an experimental therapeutic in less than 3 hrs. This time point has further advantages, as significant BSCB permeability exists between 1hr and 5 days post-injury, with peak permeability at 24hrs post-injury and recovery by 14 days post-injury^65^. This window allows MSCEv to easily penetrate the BSCB and be present during the initial activation of resident microglia and infiltration of neutrophils^66^. Inflammatory mediators TNF-α and IL-1β peak within the first 4hrs after SCI in rats^67^. Rats subjected to SCI have an intraspinal accumulation of circulating monocytes, microglia and lymphocytes peaking at 14 days post-injury. Interestingly, a single treatment of MSCEv at 3 hr was able to significantly affect the immune composition of the blood, spleen, and spinal cord across both myeloid and lymphoid cell populations. It is likely that this effect is due to MSCEv activity during the acute and sub-acute phases of injury, rather than long-term persistence and ongoing activity. While we did not define the persistence and penetration of human MSCEv in this study, other reports indicate that EVs are rapidly removed from circulation with a half-life often measured in minutes^68,69^.

Broadly, EV efficacy has been attributed to the same mechanisms as MSC, with many reports positing that EVs are more capable of crossing the blood-brain/blood-spinal cord barrier. In a recent paper^39^, we described how inflammation-stimulated MSC produce extracellular vesicles with increased anti-inflammatory properties *in vitro*, through the expression of a number of potent immunomodulating cytokines and metabolites, including COX2/PGE2, as well as micro RNA. In another study, we recently demonstrated that MSC decrease neuroinflammation in TBI via the COX2/PGE2 pathway using both a loss-of-function and a gain-of-function assay *in vivo* ^45^. The specific mechanism of action MSCEv use to modulate local and systemic inflammation is crucial to optimizing the delivery, dosage, and treatment regimen of MSCEv.

In conclusion, the data generated in this study suggests that MSCEvs are effective in attenuation of neuroinflammation and its effects on functional recovery after SCI in a rodent model. Additionally, treatment with inflammatory cytokine stimulated MSCs resulted in a modified therapeutic profile that demonstrated increased efficacy in some outcome measures.

## Electronic supplementary material

Supplemental Figures and Tables
