# Supplementary material for: Human Mesenchymal Stromal Cell-Derived Extracellular Vesicles Modify Microglial Response and Improve Clinical Outcomes in Experimental Spinal Cord Injury
Source: Sci Rep. 2018 Jan 11;8:480. doi: 10.1038/s41598-017-18867-w (PMC5764957; doi:10.1038/s41598-017-18867-w)

Supplementary Information

Human Mesenchymal Stromal Cell-Derived Extracellular Vesicles Modify Microglial Response and Improve Clinical Outcomes in Experimental Spinal Cord Injury.

Katherine A. Ruppert, PhD*, Tin T. Nguyen, MD*, Karthik S. Prabhakara, MS, Naama E. Toledano Furman, PhD, Amit K. Srivastava, PhD, Matthew T. Harting, MD, Charles S. Cox, Jr, MD, and Scott D. Olson, PhD

*Both authors contributed equally to this manuscript

Department of Pediatric Surgery, McGovern Medical School at The University of Texas Health Science Center at Houston, Houston, TX, USA

Supplemental Figure and Table Legends

Supplemental Table S1 — Locomotor Recovery Score Data

Animals treated with MSCEVs displayed significantly higher locomotor recovery scores when compared to sham and untreated SCI animals. At 5, 7 and 14 days post-injury, SCI + MSCEvs animals had significant improvement compared to untreated SCI (***,p<0.001, **, p<0.01, **,p<0.01, respectively). Treatment with MSCEVs results in significant functional improvement as early as 5 days following SCI. Values represent means +/- SEM. Sham laminectomy, n=6; SCI + Vehicle, n=6; SCI + MSCEv, n=16.

Supplemental Figure S1 — MSCEv and MSCEV+ treatment reduces the expression of RT1B in the monocytes/macrophages (cd11b/c+, cd45+, p2y12-) populations in the spinal cord. Samples of epicenter and adjacent lumbar spinal cord tissue were processed for monocyte/macrophage population ($cd11b/c^{+}$, $cd45^{+,}$ $p2y12^{-}$) and analyzed for statistical significance using a One-Way ANOVA with Tukey's correction for multiple comparisons. There is a significant decrease in this cell population from animals treated with $MSCEv^{wt}$ (*, p<0.05) and $MSCEv^{+}$ (**, p<0.01) in the injury epicenter, as well as in the lumbar section, with $MSCEv^{wt}$ (**, p<0.01) and $MSCEv^{+}$ (*, p<0.01) when compared to injured animal values. Values represent means +/- SD. SCI + Vehicle, n=3; SCI + $MSCEv^{wt}$, n=4; SCI + $MSCEv^{+}$, n=4.

Supplemental Figure S2 — MSCEv Effects on Immune Response in Blood and Spleen. Blood and spleen tissues were analyzed using flow cytometry to observe the immunomodulatory effects of $MSCEv^{wt}$ and $MSCEv^{+}$ treatment. There is a significant increase (***, p value<0.001) in blood dendritic cells in $MSCEv^{wt}$ treated animals compared to injured controls. T cell DC conjugates, which are the marker for immune reaction, are increased with either $MSCEv^{wt}$ or $MSCEv^{+}$ (a). T reg and Treg/T cell ratio increased both in the spleen and blood for groups treated with $MSCEv^{wt}$ or $MSCEv^{+}$ (b). There is significant recovery of T cytotoxic cells in the blood of $MSCEv^{wt}$ (*, p value<0.05) and $MSCEv^{+}$ (**, p value<0.01) compared to injured controls (c). All data represented are means +/- SD. Statistical significance was analyzed using unpaired t-tests. Sham laminectomy + Vehicle, n=3; SCI + Vehicle, n=3; SCI + $MSCEv^{wt}$, n=4; SCI + $MSCEv^{+}$, n=4.

Table S1. Locomotor Recovery Scores

Sham Laminectomy + Vehicle			SCI + Vehicle			SCI + MSCEVs		
Mean	SD	N	Mean	SD	N	Mean	SD	N
16	0	6	0	0	6	0.15625	0.5072393	16
16	0	6	0.3333333	0.8164966	6	0.375	0.8850612	16
16	0	6	0.3333333	0.8164966	6	0.90625	1.157854	16
16	0	6	2.166667	2.804758	6	5.21875	3.33151	16
16	0	6	7.583333	1.562583	6	9.875	1.658312	16
16	0	6	10.83333	1.505545	6	11.6875	1.973787	16
16	0	6	11.33333	1.21106	6	13.625	1.607275	16

Figure S1. MSCEv and MSCEV+ treatment reduces the expression of RT1B in the monocytes/macrophages populations in the spinal cord

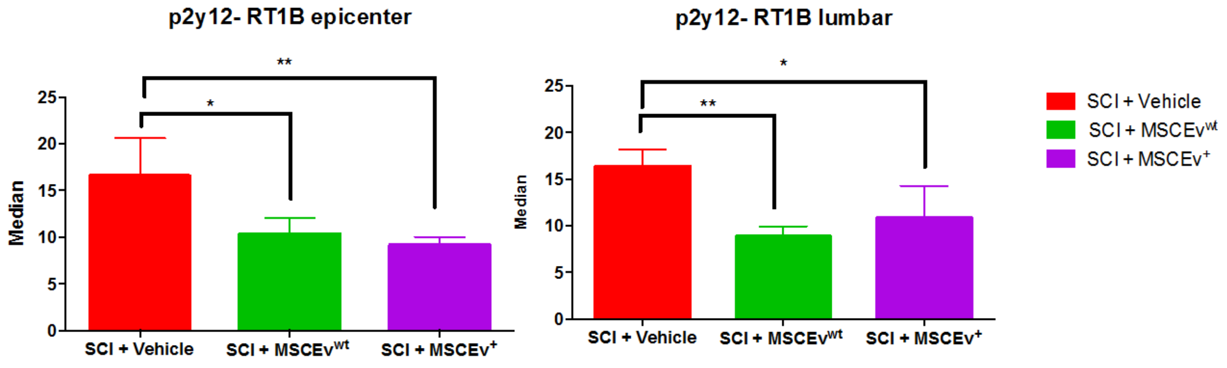

Figure S2. MSCEv Effects on Immune Response in Blood and Spleen

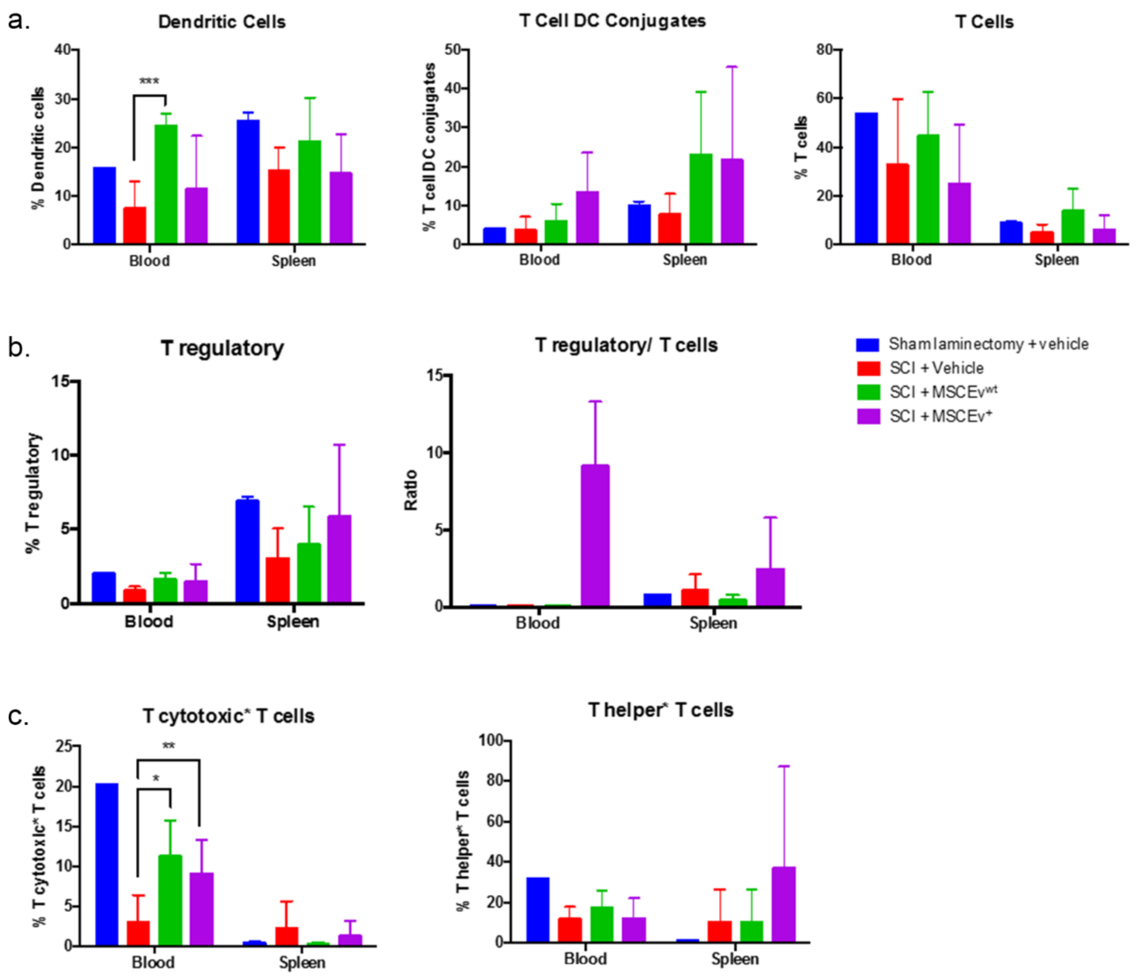

Supplement: Supplementary file 1 — Supplemental Figures and Tables [file 41598_2017_18867_MOESM1_ESM.pdf]
